# Effectiveness of direct-acting antivirals in maintenance hemodialysis patients complicated with chronic hepatitis C

**DOI:** 10.1097/MD.0000000000023384

**Published:** 2020-11-25

**Authors:** Chunhong Li, Jing Liang, Huiling Xiang, Haiyan Chen, Jie Tian

**Affiliations:** a Depatrment of Nephrology, The Third Central Clinical College of Tianjin Medical University; b Department of Nephrology, The Third Central Hospital of Tianjin; c Department of Blood Purification Center, The Second Hospital of Tianjin Medical University, Tianjin, China.

**Keywords:** chronic hepatitis C, direct-acting antivirals, hemodialysis

## Abstract

Hepatitis C virus (HCV) infection is very common in maintenance hemodialysis patients, causing high morbidity and mortality. This study aimed to evaluate the effectiveness and adverse events of direct-acting antivirals (DAAs) in maintenance hemodialysis patients complicated with chronic hepatitis C in real-world clinical practice.

In this retrospective observational study, hemodialysis patients with chronic hepatitis C infection in the Third Central Hospital of Tianjin outpatient were screened, and appropriate treatment plans were selected accordingly. Totally 25 patients diagnosed with chronic hepatitis C and treated with DAAs for 12 weeks or 24 weeks were included. The sustained virologic response (SVR) rate obtained 12 weeks post-treatment (SVR12) was evaluated. Laboratory indexes and adverse reactions during the treatment process were also assessed.

A total of 25 cases met the eligibility criteria and provided informed consent. Except for 1 patient who discontinued the treatment due to gastrointestinal bleeding, the remaining 24 cases completed the treatment cycle with 100% rapid virologic response (RVR) and 100% SVR12, with no serious adverse reactions recorded.

Maintenance hemodialysis patients complicated with chronic hepatitis C in Chinese real-world setting tolerate DAAs very well, with a viral response rate reaching 100%.

## 1 Introduction

Hepatitis C virus (HCV) infection represents a major cause of chronic liver disorders worldwide, and affects nearly 3% of the world population.^[1,2]^ The vast majority of patients are infected with HCV by blood transmission.^[3]^ Chronic kidney disease (CKD) patients are prone to HCV infection due to treatment methods such as hemodialysis and kidney transplantation.^[4]^

The Dialysis Outcomes and Practice Patterns Study (DOPPS) included 8615 hemodialysis patients in 308 hemodialysis centers, and found an average of 13.5% cases complicated with HCV infection.^[5]^ In this trial, the incidence rates differed by hemodialysis center and country, which might be related to the centers level of care and the country's economic and healthcare situations.^[5]^ Our previous survey showed an HCV infection rate in maintenance hemodialysis patients of 5.26%, which was significantly higher than that of the general population; in addition, HCV was most prevalent in individuals with bloodborne diseases.^[6]^

The above findings demonstrate that individuals undergoing hemodialysis are vulnerable to hepatitis C infection. This is compounded by the fact that hemodialysis patients are generally susceptible to infection.^[7]^ Therefore, nephrologists and nurses specialized in hemodialysis should pay increasing attention to such patients, which would improve their welfare. Previously, treatment of individuals infected with HCV was limited to interferon- and ribavirin (RBV)-containing regimens, with low cure rates and serious and unpleasant side effects.^[8]^ Due to high efficiency, low drug resistance and high safety, direct-acting antivirals (DAAs) have become the first-line treatment option for chronic HCV as recommended by international guidelines.^[9,10]^ Currently, it was shown that DAAs demonstrate good safety and efficacy in patients with renal impairment infected by HCV.^[11,12]^ However, reports assessing the application of DAAs in hemodialysis patients complicated with hepatitis C in Chinese real-world setting are scare. Therefore, the present study aimed to assess the effectiveness and adverse event of DAAs in maintenance hemodialysis patients complicated with chronic hepatitis C in China.

## 2 Patients and methods

### 2.1 Patients

Patients who visited the outpatient department of hepatology of our hospital for “chronic hepatitis C” from June 2018 to February 2020 for antiviral treatment were screened, and those with hemodialysis were enrolled. Chronic hepatitis C virus infection was defined as detectable hepatitis C virus antibodies and quantifiable serum HCV RNA by using the COBAS TaqMan HCV Kit (Roche Diagnosis Co, Ltd, Mannheim, Germany, detection limit [LLOD]: 15 IU/ml), which lasted for more than 6 months. All the enrolled patients underwent HCV antibody and HCV RNA PCR tests, and further genotyping was performed. HCV antibody was detected by enzyme-linked immunosorbent assay (ELISA).

Inclusion criteria were:

1. hepatitis C diagnosis based on current guidelines^[13]^;
2. current hemodialysis administration.

Exclusion criteria were:

1. decompensated cirrhosis,
2. serious heart disease,
3. hepatitis B,
4. HIV and
5. malignant tumors.

This observational study was approved by the Ethics Committee of the Third Central Hospital of Tianjin. Informed consent was obtained from all patients prior to study initiation.

### 2.2 Treatment plan and follow-up

Before treatment, all the patients included had undergone blood biochemistry, blood routine, coagulation function examination, alfa-fetoprotein (AFP) test, abdominal B ultrasound and liver stiffness examination. Blood samples from all patients were collected before hemodialysis on the same day. The instantaneous elastic imaging technique was performed for liver stiffness examination, and cirrhosis was considered with liver stiffness ≥12.5 kPa.^[14]^

Appropriate treatment plans were selected according to genotyping results, cirrhosis presence or not and economic factors, following AASLD guidelines.^[15]^

### 2.3 Study outcomes

Virologic response, defined as undetectable HCV RNA, was assessed at Week 4 of treatment (rapid viral response [RVR]), at the end of treatment (EOT) and at week 12 (SVR12) post-treatment. The primary efficacy endpoint was SVR12. Patients whose course of treatment had not yet reached 12 weeks after discontinuation were assessed as achieving SVR at EOT. Safety was primarily assessed by the proportion of patients who discontinued the treatment because of adverse drug reactions; in addition, patient safety over the course of treatment (drug-related or suspected adverse reactions reported by patients or their families) was assessed.

### 2.4 Data collection

Demography data, including gender, age, HCV RNA, HCV genotype, and comorbidities, were collected from medical records or interviews (patients and/or family members).

### 2.5 Statistical methods

The SPSS 25.0 software (SPSS, USA) was used for statistical analysis. Baseline and endpoint data were summarized by descriptive statistics. The t-test was performed for comparing changes in laboratory indicators. *P* < .05 was considered statistically significant.

## 3 Results

### 3.1 Baseline characteristics

There were 25 maintenance hemodialysis patients complicated with hepatitis C, including 15 males and 10 females, with an average age of 50.54 ± 11.27 years. Among them, 22 and 1 cases were genotypes 1b and 2a, respectively, and 2 were of unclear genotypes. Average viral load in HCV RNA positive patients (log10HCV RNA) was 5.53 ± 0.61 (4.36–6.91) (Table 1).

**Table 1:** Basic features of the enrolled patients.

Variables	Patients (n = 25)
Sex	
Male	15 (60.0%)
Female	10 (40.0%)
Age	50.54 ± 11.27
HCV genotype	
1b	22 (88.0%)
2a	1 (4.0%)
unclear genotyping	2 (8.0%)
Average viral load of HCV-RNA (log10 IU/ml)	5.53 ± 0.61
Treatment history of hepatitis C	2 (8.0%)
History of renal transplantation	3 (12.0%)
Cirrhosis	3 (12.0%)
complicated with hypertension / diabetes / cardiovascular disease	15 (60.0%)

Three patients with a history of kidney transplantation had lost function requiring dialysis, and were under anti-rejection drugs. In addition, 15 cases were concurrently complicated with hypertension, diabetes and cardiovascular disease. Only 2 patients had previously received interferon plus ribavirin treatment, but discontinued the medication because of significant side effects, with HCV RNA not becoming negative during the treatment. The remaining 23 patients had received no previous treatment against HCV. (Table 1).

### 3.2 Treatment plans

The initial treatment plan in the genotype 2a infected patient was Sofosbuvir 400 mg once daily/Ribavirin 400 mg twice daily; the patient showed reduced platelet levels in the fourth week of treatment, and was switched to sofosbuvir 400 mg once daily/daclatasvir 60 mg once daily for the remaining 8 weeks. The initial treatment plan in the 2 cases with unclear genotypes was Sofosbuvir 400 mg once daily/Velpatasvir 100 mg once daily for 12 weeks. Treatment plans in the remaining 22 genotype 1b infected patients were:

1. daclatasvir 60 mg once daily/asunaprevir 100 mg twice daily for 24 weeks (3 patients);
2. sofosbuvir 400 mg once daily/daclatasvir 60 mg once daily for 12 weeks (3 patients);
3. elbasvir 50 mg once daily/grazoprevir 100 mg once daily for 12 weeks (16 patients).

### 3.3 Treatment effects

Of all cases, 1 patient administered elbasvir/grazoprevir discontinued the medication because of gastrointestinal bleeding at week 9, and another treated with sofosbuvir + ribavirin was switched to sofosbuvir + daclatasvir for the last 8 weeks because of thrombocytopenia at week 4. The remaining 23 patients completed the treatment cycle as planned. HCV RNA was re-tested at 4 weeks during the treatment and 12 weeks post-treatment, respectively, and the levels were <15 IU/ml in all patients (Table 2).

**Table 2:** Negative conversion of HCV RNA in patients who completed the treatment.

		Negative conversion rate		
Treatment plan	Number of cases	RVR	EOT	SVR
SOF+DCV	4	4 (100%)	4 (100%)	4 (100%)
DCV+ASV	3	3 (100%)	3 (100%)	3 (100%)
EBR+GZR	15	15 (100%)	15 (100%)	15 (100%)
SOF+VEL	2	2 (100%)	2 (100%)	2 (100%)
Total	24	24 (100%)	24 (100%)	24 (100%)

### 3.4 Safety

Adverse reactions from treatment initiation to end were recorded, and their possible associations with the drugs were evaluated. During the treatment, 3 patients had asthenia, 2 had nausea, and 1 showed skin pruritus, which were mainly mild or moderate and well- tolerated; the planned treatment was completely applied. Only 1 patient with a history of gastric ulcer had gastrointestinal bleeding during the treatment period. Although the relationship between recurrent gastrointestinal bleeding and direct antiviral drugs could not be determined, antiviral treatment was stopped for safety reasons in this case. There were no deaths during the treatment. The data are summarized in Table 3.

**Table 3:** Adverse events occurring during treatment of patients treated with DAAs.

Adverse events	Patients, n (%) N = 25
Interruption during treatment	1 (4.0)
Any adverse event	6 (24.0)
Nausea	2 (8.0)
Itchy skin	1 (4.0)
Asthenia	3 (12.0)
Serious adverse events	0
Death	0

Changes of laboratory examination indexes during treatment and follow-up were as follows. Transaminase (aspartate transaminase [Easterbrook, #10] and alanine aminotransferase [ALT]) levels were increased in 2 patients (2 times higher than the upper limit of respective normal values). Hemoglobin fluctuation occurred in 3 patients, indicating the possibility of renal anemia, but showing a recovery trend after iron supplementation and erythropoietin dose increase. During the treatment, no significant platelet decrease was observed (Table 4).

**Table 4:** Changes of laboratory indexes during treatment.

Index	Baseline	Week 4	EOT	*t*1	*P*1	*t*2	*P*2
ALT	16.12 ± 5.78	12.46 ± 5.71	13.00 ± 6.57	2.020	.053	−0.630	.535
AST	14.21 ± 6.02	11.21 ± 5.64	12.33 ± 6.35	3.050	.006	−0.876	.570
HGB	117.67 ± 20.12	113.83 ± 19.52	112.17 ± 17.74	1.728	.097	0.754	.459
PLT	162.46 ± 10.58	160.46 ± 8.83	165.21 ± 10.44	0.235	.816	−0.928	.363

## 4 Discussion

HCV infection is very common in maintenance hemodialysis patients, causing high morbidity and mortality, and DAAs have demonstrated efficacy and safety in these patients. However, to the best of our knowledge, there is still a lack of reports assessing DAAs in Chinese Patients. Thus, this study aimed to evaluate the effectiveness and adverse events of DAAs in maintenance hemodialysis patients complicated with chronic hepatitis C in China. We found that DAAs could be successfully applied to treat maintenance hemodialysis patients complicated with chronic hepatitis C, with an impressive viral response rate of 100% (both RVR and SVR) and no overt adverse reactions.

Chronic hepatitis C represents a common bloodborne disease, and the HCV infection rate is higher in hemodialysis patients compared with the general patient population due to factors such as blood transfusion and hemodialysis treatment.^[16,17]^ As an additional source of infection, HCV in hemodialysis patients not only further increases the exposure risk of other patients and medical workers, but also causes kidney deterioration and liver diseases in the patients themselves, significantly increasing all-cause mortality. Indeed, liver disease-related mortality, cardiovascular mortality and infection-related mortality are all significantly increased in hemodialysis patients complicated with HCV infection.^[18]^ Therefore, antiviral treatment must be timely administered to hemodialysis patients with HCV infection.^[19]^ The Clinical Guidelines for Hepatitis C in CKD Patients published by Kidney Disease: Improving Global Outcomes (KDIGO) in 2018 proposed that new infections should be detected and treated as early as possible.^[20]^ Previously, the serious side effects of interferon-based anti-HCV treatment has limited its application in end-Stage Renal Disease (ESRD) patients.^[8]^ DAAs are effective in hemodialysis patients with chronic hepatitis C.^[21]^ The availability of safe and efficient DAAs provides novel opportunities, including the transplantation of kidneys from HCV-infected kidney donors, which could significantly affect patient care with favorable long-term outcomes.^[22]^ However, related studies carried out in Chinese patients are rare.

It was shown that the most common HCV genotype in China is type 1, followed by types 2, 3 and 6; in terms of subtypes, genotype 1b is most common in Chinese individuals, accounting for 56.8% of all HCV infections.^[23]^ As shown above, genotype 1b accounted for 88% of all enrolled patients, which was overtly higher than the above figure, but confirming its predominance. The discrepancy may be due to the small sample of this trial.

DAAs directly act on the protease and RNA polymerase of HCV as well as other important mediators of viral replication, effectively inhibiting viral replication. The Guidelines for the prevention and treatment of hepatitis C (2015 version)^[24]^ pointed out that the first therapeutic choice for hepatitis C patients with renal damage should be interferon-free oral antiviral drugs. In the present study, one patient was initially treated with ribavirin-containing antiviral regimen, but thrombocytopenia occurred during the treatment, which was switched to a DAA regimen, and SVR was achieved.

Since DAAs were approved for the Chinese market in 2017, they have not been widely used in hemodialysis patients due to high cost. In addition, studies evaluating DAAs in China are scarce. In order to further improve the cure rate in hepatitis C and reduce the odds of viral transmission, the Tianjin Healthcare Security Administration has approved the policy of pay per person for the treatment of chronic hepatitis C since May 2018, so that more individuals could receive treatment, especially hemodialysis patients. The current findings may help promote the use of these medications by Chinese hemodialysis patients complicated with HCV.

Currently, most approved DAAs such as elbasvir, grazoprevir, daclatasvir, asunaprevir, paritaprevir and ombitasvir are not eliminated by the kidney, and there is no need for dose adjustment even in severe chronic kidney disease or hemodialysis patients.^[25]^ In this study, 3 genotype 1b infected patients were treated with the daclatasvir/asunaprevir regimen, which was effective and safe, with an SVR reaching 100%, corroborating Kawakami et al..^[26]^ Suda et al. reported an SVR for the elbasvir/grazoprevir regimen of 96.7% (22/23),^[27]^ while the SVR in the 11 patients treated with the latter regimen was 100% in this study; this rate was slightly higher than that reported by Suda and colleagues, likely because there were more cirrhosis patients in the latter study.

Among currently approved DAAs, sofosbuvir is the only drug mainly eliminated by the kidney, with 72% renal elimination.^[28]^ Guidelines for Hepatitis C treatment published by the European Association for the Study of the Liver also suggested that sofosbuvir should be used cautiously in patients with severe renal insufficiency and ESRD with eGFR < 30 ml/(minutes·1.73 m^2^), with no recommended dose.^[19]^ However, studies demonstrated sustained virological responses reaching 88% to 96% for sofosbuvir-based DAA treatments in CKD patients with eGFR < 30 ml/(minutes·1.73 m^2^).^[29,30]^ In addition, it was pointed out that sofosbuvir/vipatavir treatment (pangenotype DAA regimen) could be selected for patients with CKD 5D stage without dosage adjustment according to the Guidelines for the prevention and treatment of hepatitis C (2019 version).^[31]^ In this study, 3 patients were treated with sofosbuvir/daclatasvir, and 2 with sofosbuvir/velpatasvir, and all achieved SVR, with no serious adverse effects or abnormal laboratory indicators during the treatment, in agreement with the above studies. In addition, hemodialysis patients have more complications, such as renal hypertension and renal anemia, and other drugs must be administered concurrently. There were 3 patients with renal allograft dysfunction in this study, and anti-rejection drugs such as tacrolimus and cyclosporine were provided in combination with DAAs. This study showed that DAAs are well tolerated, with no serious adverse events and unaltered laboratory indexes of toxicity. These findings support the application of DAAs in Chinese hemodialysis patients complicated with HCV.

The main limitation of this study was its small sample size. Consequently, the safety of sofosbuvir in hemodialysis patients could not be assessed. Therefore, the number of included patients should be further increased, and different liver functions should also be determined to further evaluate DAA toxify. Finally, the effects of other drugs on the blood concentrations of DAAs were not evaluated, which requires further investigation.

In conclusion, this study showed that DAA therapy has good effectiveness in hemodialysis patients with HCV. These findings support the application of DAAs in Chinese hemodialysis patients complicated with HCV. Due to the limited sample size, further studies are warranted to confirm the present findings.

## Author contributions

XXXXX.
